# Canine hepacivirus is not associated with chronic liver disease in dogs

**DOI:** 10.1111/jvh.12150

**Published:** 2013-08-12

**Authors:** N. H. Bexfield, P. J. Watson, J. Heaney, J. L. Heeney, L. Tiley

**Affiliations:** ^1^ Department of Veterinary MedicineUniversity of CambridgeCambridgeUK

**Keywords:** aetiology, dog, hepatitis, respiratory, RNA virus

## Abstract

Canine hepacivirus (CHV) has recently been identified in liver and respiratory tract samples from dogs, and comparative phylogenetic analysis has confirmed it to be the closest genetic relative of hepatitis C virus (HCV) described to date. CHV offers great potential as a model system for HCV, but only if the underlying processes of infection and pathogenesis are similar for both viruses. However, it is not yet clear if CHV is hepatotrophic. Canine chronic hepatitis (CH) is a common and usually idiopathic disease that shares similar histological features to that of HCV infection of humans. To date, no study has attempted to determine whether CHV is involved in the aetiology of liver disease in dogs. We employed two nested PCR assays, using primers targeting regions of the helicase domain of CHV NS3, to identify viral nucleic acids in liver samples from 100 dogs with CH of unknown cause in the UK. We also used a sensitive luciferase immunoprecipitation system (LIPS) assay to screen serum samples from these dogs for the presence of anti‐CHV antibodies. Surprisingly, there was no evidence of exposure to, or a carrier state of, CHV in this large cohort, suggesting that the virus is not associated with CH in UK dogs. Future work, including transmission studies, is required to understand the pathogenesis of CHV in canids before it can be proposed as a surrogate model for HCV‐induced liver disease in man.

**Abbreviations**
CH: Chronic hepatitis
CHV: Canine hepacivirus
GBV‐B: GB virus B
HAI: Histology activity index
HCV: Hepatitis C virus
LIPS: Luciferase immunoprecipitation system
LU: Light unit
NPHV: Non‐primate hepacivirus
SD: Standard deviation
UTR: Untranslated region

Canine hepacivirus (CHV), recently identified in respiratory tract samples from dogs with respiratory illness in the USA, was also detected in liver tissue from dogs with unexplained gastrointestinal illness 1. *In situ* hybridisation confirmed the presence of viral RNA predominantly in cytoplasm of hepatocytes. Molecular characterization of CHV suggested its genome is at least 9195 nucleotides and encodes a 2942 amino acid polyprotein and a short 5′ untranslated region (UTR) 1. Among hepaciviruses, CHV was found to be more similar throughout the genome to HCV than to GB virus B (GBV‐B) 1. Comparative phylogenetic analysis of CHV confirmed it to be the closest genetic relative of HCV described to date 1.

An estimated three per cent of the world's population is chronically infected with HCV, with and more than 350 000 people dying from HCV‐related liver diseases every year 3. However, efforts to understand human HCV infection have been hampered by the absence of suitable animal models other than the chimpanzee and, until recently, its inability to replicate in cell culture 4. Despite CHV being identified in low levels in canine liver tissue, it is unclear if this virus is hepatotrophic. If CHV is associated with liver disease in dogs, the ability to study hepacivirus pathogenesis, immunity and treatment in a more tractable animal model would dramatically alter the progress of HCV research 2.

Chronic hepatitis (CH) is the most frequently reported canine liver disease and has a postmortem prevalence in the UK of 12% 9. As the aetiology of most cases of canine CH remains unknown 10, and the disease shares histologically features with that of HCV infection in humans 12, CHV is a candidate aetiological agent of CH. Studies have failed to identify HCV in canine CH 10; however, to date, no study has reported CHV in dogs with CH.

Following the initial identification of CHV in dogs 1, several nonprimate animal species have since been screened for the presence of anti‐CHV antibodies 16. A sensitive luciferase immunoprecipitation system (LIPS) assay 17 was used with the evolutionary conversed CHV helicase protein as the target antigen. Samples of 36 from 103 horses were immunoreactive, and viral genomic RNA was present in eight seropositive animals and none of the seronegatives. Complete genome sequence analysis revealed 14% (range 6.4–17.2%) nucleotide sequence divergence, with most changes occurring at synonymous sites 16. These viruses have been named nonprimate hepaciviruses (NPHV 1–8). Interestingly, in this same study, none of 80 serum samples from dogs were seropositive, although the health status of these animals was not known.

The aim of this study was to test the hypothesis that CHV is the aetiological agent of canine CH by detecting viral RNA in affected liver tissue and/or demonstrating the presence of anti‐CHV antibodies. To achieve this aim, we used two nested PCRs to amplify CHV in liver tissue from a large cohort of dogs with CH, and also a LIPS assay to determine whether dogs with CH have anti‐CHV antibodies.

## Materials and Methods

### Sample details

Liver and blood samples were obtained from 100 dogs with a histological diagnosis of CH according to established criteria 14. To maximize the likelihood of detecting CHV, cases were selected where liver histology demonstrated changes particularly suggestive of a viral aetiology, primarily the presence of a lymphocyte‐rich inflammatory cell infiltrate 12. All dogs were resident in the UK. There were a total of twenty different pedigree breeds and also crossbreed dogs, with eight represented by five or more dogs including the Labrador retriever (*n* = 12), English springer spaniel (*n* = 11), crossbreed (*n* = 11), English cocker spaniel (*n* = 8), Dobermann pinscher (*n* = 7), American cocker spaniel (*n* = 5), Cairn terrier (*n* = 5) and Yorkshire terrier (*n* = 5). No dog had received immunosuppressive or antiviral therapy within the preceding 4 weeks. Liver tissue was obtained when dogs underwent a liver biopsy as part of their investigations, and blood samples consisted of that remaining after routine diagnostic testing. All samples were collected with written and informed owner consent and with the approval from the Institutional Ethics and Welfare Committee. Liver tissue was immediately frozen at −80 °C, or collected into RNAlater (Life Technologies Ltd, Paisley, Renfrewshire, UK), stored at 4 °C for up to 24 h then frozen at −80 °C. Blood samples were collected without anticoagulant, separated within 6 h of collection, and the serum frozen at −80 °C within 48 h.

### Nested PCR

Total RNA was extracted from 100 mg of liver tissue using TRIzol reagent (Life Technologies Ltd) according to the manufacturer's instructions. After reverse transcription using random primers, resulting cDNA was used in two separate nested PCRs for detection of the helicase domain of CHV NS3. Each pair of PCR primers was optimized for primer annealing temperature.

The first PCR used previously published primers targeting nucleotide positions 4186–4636 of CHV (GenBank accession number JF744991.1) 1, with primer for the first round (Chcv‐0F1: 5′‐TCCACCTATGGTAAGTTCTTAGC‐3′ and Chcv‐0R1: 5′‐ACCCTGTCATAAGGGCGTC‐3) and the second round (Chcv‐0F2: 5′‐CCTATGGTAAGTTCTTAGCTGAC‐3′ and Chcv‐0R2: 5′‐CCTGTCATAAGGGCGTCCGT‐3′). First‐round amplification consisted of 30 cycles of 95 °C for 20 s, 58 °C for 20 s and 72 °C for 40 s in an automated thermal cycler (Bio‐Rad iCycler, Bio‐Rad Laboratories, Hemel Hempstead, Hertfordshire, UK). Second‐round amplification using real‐time PCR and SYBR green chemistry (Platinum SYBR green qPCR supermix‐UDG; Life Technologies Ltd) consisted of 45 cycles of 95 °C for 10 s, 58 °C for 15 s and 60 °C for 15 s in an automated thermal cycler (Rotor‐Gene 6000, Corbett Life Science/Qiagen, Cambridge, Cambridgeshire, UK). Positive controls for first‐ and second‐round amplification consisted of a 450 base synthetic oligonucleotide (GeneART; Life Technologies Ltd) containing the region of the CHV genome amplified by the outer primers. The gene was cloned into the vector pMA‐T with plasmid DNA used as the template for PCR and also sequenced to confirm identity. The sensitivity of each nested PCR was calculated using triplicate ten‐fold dilutions of positive control from 1x10^8^ to 1x10^0^ genome copies per reaction, while observing the lowest dilution which produced fluorescence above background followed by an exponential increase in fluorescence. Standard precautions were taken to avoid PCR contamination, and no false positives were observed in negative controls.

The second PCR was a consensus PCR using primers designed by aligning conserved regions of all available CHV sequences in the GenBank database (NCBI, http://www.ncbi.nlm.nih.gov/, accession JF744991.1 to JF744996.1), with primers for the first round (Chv‐NS3F1: 5′‐ GCCATAGCACAGACTCCACA‐3′ and Chv‐NS3R1: 5′‐ AAGGGTATGTCACCGCTCTG‐3′) and second round (Chv‐NS3F2: 5′‐CCTATGGTAAGTTCTTAGCTGAC‐3′ and Chv‐NS3R2: 5′‐CGATGTTAGGATGAGGGACAG‐3′). PCR conditions for first‐ and second‐round amplification were as described previously, with the exception of annealing temperature of 61 °C. The positive control consisted of a 171 base synthetic oligonucleotide synthesized as previously described and sequenced to confirm identity.

### LIPS assay

A LIPS assay was used to screen serum sample from dogs with CH for the presence of anti‐CHV antibodies. To produce the *Renilla* luciferase (Ruc)‐CHV helicase antigen fusion construct, a template for the NS3 helicase‐coding sequence of CHV was generated from a respiratory sample of a dog as previously described 16. The primer adapter sequences used to clone the CHV protein fragment were as follows: 5′‐GAGGGATCCATACACTTCGCAGATATG‐3′ and 5′‐GAGCTCGAGTCAGGTGTTACAGTCAGTAAC‐3′. The protein fragment was subcloned downstream of Ruc using the pREN2 vector 18. DNA sequencing was used to confirm the integrity of the DNA construct. Plasmid DNA was prepared, and following transfection of mammalian expression vectors, crude protein extract was obtained as described for use as antigen 19. Canine sera were processed in a 96‐well format at room temperature as previously described 19. Light units (LUs) were measured in a Berthold LB 960 Centro microplate luminometer (Berthold Technologies, Oak ridge, TN, USA) using coelenterazine substrate mix (Promega, Madison, WI, USA). All LU data were obtained from the average of two separate experiments. GraphPad Prism software (GraphPad Software Inc., La Jolla, CA, USA) was used for statistical analysis of LIPS data. As positive controls, serum sample from four horses previously determined to have low (*n* = 2), medium (*n* = 1) and high levels (*n* = 1) of anti‐CHV NS3 helicase antibodies where analysed in parallel to the canine samples. To classify samples as positive, a cut‐off limit derived from the combined value of the mean value plus three standard deviations (SD) of the replica negative control samples containing only buffer, Ruc‐extract and protein A/G beads was used.

## Results

### Nested PCR for detection of CHV in canine liver tissue

**Figure 1:**
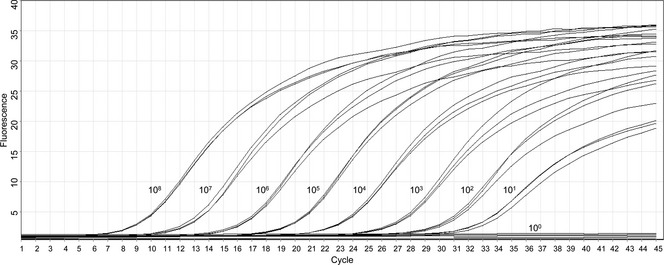
Results of real‐time PCR to determine the sensitivity of the first nested PCR. The triplicate dilutions of the positive control, in copies per reaction, are depicted. *Y*‐axis, normalized fluorescence; *X*‐axis, cycle number.

**Figure 2:**
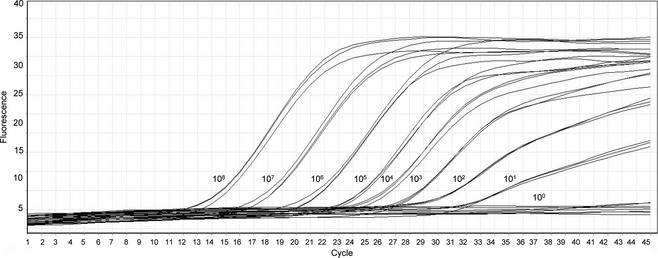
Results of real‐time PCR to determine the sensitivity of the second nested PCR. The triplicate dilutions of the positive control, in copies per reaction, are depicted. *Y*‐axis, normalized fluorescence; *X*‐axis, cycle number.

RNA extracted from liver tissue of 100 dogs with histologically confirmed CH was converted to cDNA and used as a template in two nested PCRs targeting regions of the helicase domain of CHV NS3. Samples from all 100 dogs were negative for CHV in both nested PCRs. The sensitivity of the first (Fig. 1) and second PCRs (Fig. 2) was ten genome copies per reaction.

### LIPS assay

**Figure 3:**
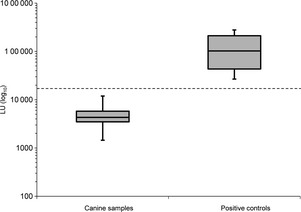
Box and whiskers plot showing the mean results of an LIPS assay for the detection of anti‐NS3 CHV antibodies in 100 dogs with chronic hepatitis (canine samples) and from known positive equine samples (positive controls). The *Y*‐axis depicts the antibody titres in light units (LU) on a log_10_ scale. The boxes represent the interquartile intervals from the 25th to 75th percentiles, and the solid horizontal bars through the boxes represent the medians. The capped vertical bars represent the minimum and maximum of all data. The horizontal dotted line shows the cut‐off limit derived from the combined value of the mean value plus 3 standard deviations (SD) of negative control samples.

The median LU from horses previously determined to have low, medium and high levels of anti‐CHV helicase antibodies was 102 503 (range 26 757–278 974). A cut‐off limit of 17 520 LU was used to classify samples as positive. Serum samples from 100 dogs with histologically confirmed CH were tested, and all were negative (median 4306; range 1445–11 916 LU) (Fig. 3).

## Discussion

This is the first study which aimed to determine whether there is an association of CHV RNA and anti‐CHV antibodies in dogs with CH. Canine CH, a common liver disease in the UK 9, shares histologically features with that of HCV infection in humans 12. The aetiology of the majority of cases remains unknown 10, despite demonstration of a transmissible agent capable of causing CH in dogs in the UK more than 25 years ago 24. Studies using a candidate virus approach to identify such an agent, including HCV, have been repeatedly unsuccessful 10.

Despite the previous report of low levels of CHV RNA in liver tissue of dogs with unexplained gastrointestinal illness, and the detection of virus in canine hepatocytes by *in situ* hybridisation 1, we were unable to demonstrate CHV in liver tissue from any of 100 dogs with CH. It is not clear if the dogs in this initial report also had respiratory tract disease, and if so, it is possible that viral antigen was ingested in sputum, passed across a compromised gastrointestinal tract and transported to the liver in the hepatic portal vein. CHV may be an innocent bystander, as was the case for GBV‐C, initially proposed as a unique hepatitis virus in humans 28. Indeed, the application of new sequence‐independent amplification strategies and sequencing technologies has lead to the discovery of numerous new viruses, which may or not have disease relevance 29. Future studies are required to define the pathogenesis of CHV before it can be adopted as a surrogate model for HCV‐induced liver disease in this species. For HCV, the ability to cause viral hepatitis was only definitively proven by inoculating primates with RNA transcripts from synthetic DNA recombinants 30. Similar inoculation experiments may be necessary in dogs before the pathogenesis of CHV can be elucidated. Our failure to detect CHV may also reflect sequence divergence that confounds PCR. Having said that CHV sequences identified to date have shown relatively little genetic diversity 1. The publication of additional CHV genomes may enable alternative regions of sequence conservation to be selected for primer design. Moreover, the sequence of CHV circulating among dogs in the UK may be distinct from that present in the USA.

For viral detection, we employed two nested PCRs, one of which used previously published primers to amplify a region of the helicase domain of CHV NS3 1. To date, the sequences of one full and five partial CHV genomes have been published, and only the full genome contains the region amplified by these primers. Despite the apparent limited genetic diversity of CHV 1, sequence variation in the primer binding region could possibly have excluded PCR amplification of CHV sequences in the cohort of dogs we tested herein. For this reason, a second nested consensus PCR was designed to amplify a conserved region of all published CHV sequences, also located in the helicase domain of the NS3 gene. To maximize the likelihood of detecting CHV, cases were selected where liver histology demonstrated changes, which, particularly suggestive of a viral aetiology 12. In humans with HCV‐induced CH, increased levels of intrahepatic virus are associated with a higher histology activity index (HAI) score 32; therefore, canine cases with a higher HAI score were also chosen. Despite a sensitivity of 10 viral copies per reaction, we were not able to detect CHV in any liver sample. This high sensitivity is likely due to the fact that the PCR assays were designed to amplify a single sequence, with this sequence then used as the positive control. It cannot be excluded that CHV provokes acute liver damage, is cleared, but immunopathologically provokes a self‐perpetuating chronic liver disease. Therefore, further studies using tissues from dogs with acute disease are warranted. In human HCV infection, liver tissue contains a substantially higher titre of viral transcripts compared with blood 35, and so we did not attempt to amplify CHV in canine blood samples.

To further study the role of CHV in canine liver disease, we also employed a recently developed LIPS assay 16, to determine whether dogs with CH had anti‐CHV antibodies. Serology is more tolerant of sequence divergence and has the additional benefit of detecting resolved as well as acute or active infections. Due to the possibility that different CHV variants would be genetically diverse, an antigen from the helicase domain of NS3 was used, as this is the most conserved viral protein and one that shows documented antigenicity in HCV 38. However, we were unable to detect anti‐CHV antibodies in any of the 100 dogs tested, suggesting a lack of previous exposure to this virus. These results are consistent with those of a previous study which also failed to detect anti‐CHV antibodies in 80 dogs 16, although the health status of these animals was unknown. As the duration of immunity following CHV infection has not been studied, it is, however, possible that dogs clear CHV relatively rapidly and also become seronegative.

Important differences exist between CHV and HCV, which may have biological significance and explain potential differences in pathogenicity. Most notably, these include the apparent absence of certain micro(mi)RNA‐binding sequences in CHV 1. The 5′UTR of HCV contains two miR‐122‐binding sites that are highly conserved among all genotypes and facilitate replication in liver cells 39. Dogs encode identical miR‐122s, and these are highly expressed in the liver, but the absence of the binding site of miR‐122 in CHV suggests that the interaction may not be a feature of CHV infection. It remains to be determined whether the unique RNA structure in CHV allows the virus to replicate in a manner independent of miR‐122 and/or influences tissue tropism.

In conclusion, the data obtained in the present study do not support the role of CHV in the aetiology of canine CH. However, the absence of CHV infection in dogs from the UK might not represent the global ecology of the virus. Moreover, no studies to date have attempted to identify CHV in dogs with respiratory disease in the UK. Additional work must be performed to determine the role, if any, of the virus in different geographical locations and to confirm whether the virus is an endemic canine pathogen. Despite high sequence similarities in their envelope proteins, CHV and HCV may bind to different host cell receptors, resulting in very different tissue targets‐one in the respiratory tract and the other in the liver. This does, however, provide a unique opportunity to compare the molecular and cellular basis for those differences. Additional work must be carried out to understand the biology and pathogenesis of CHV in canids and to determine whether it has a role as a surrogate model system for HCV infection in humans.

## Conflict of Interest

This study was funded by a Wellcome Trust Research Training Fellowship to Nicholas H. Bexfield, grant number WT088619MA.

## References

[1] Kapoor A, Simmonds P, Gerold G*et al* Characterization of a canine homolog of hepatitis C virus. Proc Natl Acad Sci U S A 2011; 108(28): 11608–11613 21610165 10.1073/pnas.1101794108 PMC3136326

[2] Bukh J, Hepatitis C homolog in dogs with respiratory illness. Proc Natl Acad Sci USA 2011; 108(31): 12563–12564 21768355 10.1073/pnas.1107612108 PMC3150949

[3] WHO , Hepatitis C. Surveillance and Control. Fact sheet N°164: World Health Organization, Switzerland, 2012

[4] Bukh J, Meuleman P, Tellier R*et al* Challenge pools of hepatitis C virus genotypes 1‐6 prototype strains: replication fitness and pathogenicity in chimpanzees and human liver‐chimeric mouse models. J Infect Dis 2010; 201(9): 1381–1389 20353362 10.1086/651579 PMC2941994

[5] Lindenbach BD, Evans MJ, Syder AJ*et al* Complete replication of hepatitis C virus in cell culture. Science 2005; 309(5734): 623–626 15947137 10.1126/science.1114016

[6] Lohmann V, Korner F, Koch J, Herian U, Theilmann L, Bartenschlager R. Replication of subgenomic hepatitis C virus RNAs in a hepatoma cell line. Science 1999; 285(5424): 110–113 10390360 10.1126/science.285.5424.110

[7] Dolgin E. Research technique: the murine candidate. Nature 2011; 474(7350): S14–S15 21666729 10.1038/474S14a

[8] Murray CL, Rice CM. Turning hepatitis C into a real virus. Annu Rev Microbiol 2011; 65: 307–327 21682640 10.1146/annurev-micro-090110-102954

[9] Watson PJ, Roulois AJ, Scase TJ, Irvine R, Herrtage ME. Prevalence of hepatic lesions at post‐mortem examination in dogs and association with pancreatitis. J Small Anim Prac 2010; 51(11): 566–572 10.1111/j.1748-5827.2010.00996.x 20973784

[10] Bexfield NH, Andres‐Abdo C, Scase TJ, Constantino‐Casas F, Watson PJ. Chronic hepatitis in the English springer spaniel: clinical presentation, histological description and outcome. Vet Rec 2011; 169(16): 415. doi:10.1136/vr.d4665 21852307 10.1136/vr.d4665 PMC3361955

[11] Boomkens SY, Slump E, Egberink HF, Rothuizen J, Penning LC. PCR screening for candidate etiological agents of canine hepatitis. Vet Microbiol 2005; 108(1–2): 49–55 15917133 10.1016/j.vetmic.2005.03.003

[12] Bateman AC. Patterns of histological change in liver disease: my approach to ‘medical’ liver biopsy reporting. Histopathology 2007; 51(5): 585–596 17617216 10.1111/j.1365-2559.2007.02765.x

[13] Ishak KG. Pathologic features of chronic hepatitis. A review and update. Am J Clin Pathol 2000; 113(1): 40–55 10631857 10.1309/42D6-W7PL-FX0A-LBXF

[14] Van den Ingh TSGAM, Van Winkle TJ, Cullen JM, Charles JA, Desmet VJ. Morphological classification of parenchymal disorders of the canine and feline liver: 2 Hepatocellular death, hepatitis and cirrhosis In: Rothuizen J, Bunch SE, Charles JA*et al* eds. WSAVA Standards for Clinical and Histological Diagnosis of Canine and Feline Liver Disease, 1 edn. Philadelphia, PA: Saunders Elsevier, 2006: 85–102

[15] Guido M, Mangia A, Faa G. Chronic viral hepatitis: the histology report. Dig Liver Dis 2011; 43(Suppl 4): S331–S343 21459339 10.1016/S1590-8658(11)60589-6

[16] Burbelo PD, Dubovi EJ, Simmonds P*et al* Serology‐enabled discovery of genetically diverse hepaciviruses in a new host. J Virol 2012; 86(11): 6171–6178 22491452 10.1128/JVI.00250-12 PMC3372197

[17] Burbelo PD, Ching KH, Bren KE, Iadarola MJ. Searching for biomarkers: humoral response profiling with luciferase immunoprecipitation systems. Expert Rev Proteomics 2011; 8(3): 309–316 21679112 10.1586/epr.11.23 PMC3818131

[18] Burbelo PD, Goldman R, Mattson TL. A simplified immunoprecipitation method for quantitatively measuring antibody responses in clinical sera samples by using mammalian‐produced Renilla luciferase‐antigen fusion proteins. BMC Biotechnol 2005; 5: 22 16109166 10.1186/1472-6750-5-22 PMC1208859

[19] Burbelo PD, Ching KH, Klimavicz CM, Iadarola MJ. Antibody profiling by Luciferase Immunoprecipitation Systems (LIPS). J Vis Exp 2009; (32): pii. 1549 19812534 10.3791/1549 PMC3164068

[20] Burbelo PD, Bren KE, Ching KH*et al* LIPS arrays for simultaneous detection of antibodies against partial and whole proteomes of HCV. HIV and EBV. Mol BioSyst 2011; 7(5): 1453–1462 21336381 10.1039/c0mb00342e PMC3430135

[21] Burbelo PD, Ching KH, Mattson TL, Light JS, Bishop LR, Kovacs JA. Rapid antibody quantification and generation of whole proteome antibody response profiles using LIPS (luciferase immunoprecipitation systems). Biochem Biophys Res Commun 2007; 352(4): 889–895 17157815 10.1016/j.bbrc.2006.11.140

[22] Poldervaart JH, Favier RP, Penning LC, van den Ingh TS, Rothuizen J. Primary hepatitis in dogs: a retrospective review (2002‐2006). J Vet Intern Med 2009; 23(1): 72–80 19175724 10.1111/j.1939-1676.2008.0215.x

[23] Watson PJ. Chronic hepatitis in dogs: a review of current understanding of the aetiology, progression, and treatment. Vet J 2004; 167(3): 228–241 15080872 10.1016/S1090-0233(03)00118-7

[24] Jarrett WF, O'Neil BW. A new transmissible agent causing acute hepatitis, chronic hepatitis and cirrhosis in dogs. Vet Rec 1985; 116(24): 629–635 4024428 10.1136/vr.116.24.629

[25] Jarrett WF, O'Neil BW, Lindholm I. Persistent hepatitis and chronic fibrosis induced by canine acidophil cell hepatitis virus. Vet Rec 1987; 120(10): 234–235 3576923 10.1136/vr.120.10.234

[26] Chouinard L, Martineau D, Forget C, Girard C. Use of polymerase chain reaction and immunohistochemistry for detection of canine adenovirus type 1 in formalin‐fixed, paraffin‐embedded liver of dogs with chronic hepatitis or cirrhosis. J Vet Diagn Invest 1998; 10(4): 320–325 9786518 10.1177/104063879801000402

[27] Rakich PM, Prasse KW, Lukert PD, Cornelius LM. Immunohistochemical detection of canine adenovirus in paraffin sections of liver. Vet Pathol 1986; 23(4): 478–484 3018984 10.1177/030098588602300419

[28] Simons JN, Leary TP, Dawson GJ*et al* Isolation of novel virus‐like sequences associated with human hepatitis. Nat Med 1995; 1(6): 564–569 7585124 10.1038/nm0695-564

[29] Bexfield N, Kellam P. Metagenomics and the molecular identification of novel viruses. Vet J 2011; 190(2): 191–198 21111643 10.1016/j.tvjl.2010.10.014 PMC7110547

[30] Kolykhalov AA, Agapov EV, Blight KJ, Mihalik K, Feinstone SM, Rice CM. Transmission of hepatitis C by intrahepatic inoculation with transcribed RNA. Science 1997; 277(5325): 570–574 9228008 10.1126/science.277.5325.570

[31] Yanagi M, Purcell RH, Emerson SU, Bukh J. Transcripts from a single full‐length cDNA clone of hepatitis C virus are infectious when directly transfected into the liver of a chimpanzee. Proc Natl Acad Sci U S A 1997; 94(16): 8738–8743 9238047 10.1073/pnas.94.16.8738 PMC23104

[32] Adinolfi LE, Andreana A, Utili R, Zampino R, Ragone E, Ruggiero G. HCV RNA levels in serum, liver, and peripheral blood mononuclear cells of chronic hepatitis C patients and their relationship to liver injury. Am J Gastroenterol 1998; 93(11): 2162–2166 9820390 10.1111/j.1572-0241.1998.00613.x

[33] Coelho‐Little E, Jeffers LJ, Bartholomew M, Reddy KR, Schiff ER, Dailey PJ. Correlation of HCV‐RNA levels in serum and liver of patients with chronic hepatitis C. J Hepatol 1995; 22(2): 248–249 7790715 10.1016/0168-8278(95)80437-4

[34] Gordon SC, Kodali VP, Silverman AL*et al* Levels of hepatitis C virus RNA and liver histology in chronic type C hepatitis. Am J Gastroenterol 1994; 89(9): 1458–1461 8079919

[35] Terrault NA, Dailey PJ, Ferrell L*et al* Hepatitis C virus: quantitation and distribution in liver. J Med Virol 1997; 51(3): 217–224 9139087

[36] White PA, Pan Y, Freeman AJ*et al* Quantification of hepatitis C virus in human liver and serum samples by using LightCycler reverse transcriptase PCR. J Clin Microbiol 2002; 40(11): 4346–4348 12409428 10.1128/JCM.40.11.4346-4348.2002 PMC139708

[37] McGuinness PH, Bishop GA, Painter DM, Chan R, McCaughan GW. Intrahepatic hepatitis C RNA levels do not correlate with degree of liver injury in patients with chronic hepatitis C. Hepatology 1996; 23(4): 676–687 8666317 10.1002/hep.510230404

[38] Burbelo PD, Kovacs JA, Ching KH*et al* Proteome‐wide anti‐hepatitis C virus (HCV) and anti‐HIV antibody profiling for predicting and monitoring the response to HCV therapy in HIV‐coinfected patients. J Infect Dis 2010; 202(6): 894–898 20684729 10.1086/655780 PMC2924471

[39] Jangra RK, Yi M, Lemon SM. Regulation of hepatitis C virus translation and infectious virus production by the microRNA miR‐122. J Virol 2010; 84(13): 6615–6625 20427538 10.1128/JVI.00417-10 PMC2903297

[40] Jopling CL, Yi M, Lancaster AM, Lemon SM, Sarnow P. Modulation of hepatitis C virus RNA abundance by a liver‐specific MicroRNA. Science 2005; 309(5740): 1577–1581 16141076 10.1126/science.1113329

